# Supplementary material for: Novel Freshwater Ascomycetes from Spain
Source: J Fungi (Basel). 2022 Aug 14;8(8):849. doi: 10.3390/jof8080849 (PMC9410038; doi:10.3390/jof8080849)
Supplement: Supplementary file 1 [file jof-08-00849-s001.zip › jof-1826828-supplementary.pdf]

Table S1. Strains and sequence accession numbers included in this study.

Taxon	Strain number[1]	GenBank accession number				
		LSU[3]	ITS	*tub2*	*rpb2*	*tef1*
Allocucurbitaria botulispora	CBS 234.92 [T]	LN907416	LT592932	LT593001	LT593070	
Amniculicola aquatica	MFLUCC 16-1123 [T]	MK106096				MK109800
A. guttulata	MFLUCC 18-1038 [T]	MN913712	MT627726			MT954389
A. immersa	CBS 123083	NG_056964				GU456273
A. lignicola	CBS 123094 [T]	MH874798				GU456278
A. longissima	CB L22	GU266240	AY204596			
A. longissima	CCM-F11791	AY204594	AY204597			
A. microspora	**FMR 17946**[2] **CBS 148919** [T]	**ON008450**	**ON006460**			**ON101726**
A. parva	CBS 123092 [T]	FJ795497	MH863272			GU349065
Arthopyrenia salicis	CBS 368.94	KF443410				
Chaetomella endophytica	SDBR-CMU300 [T]	MG406984	MG406985			
C. raphigera	BPI 843541 [T]	AY487077	AY487076			
C. raphigera	CBS 130.64	MH870019	MH858390			
C. raphigera	BPI 843551	AY487086	AY487085			
C. zambiensis	CBS 137978 [T]	MH878619	NR_156280			
Cucurbitaria berberidis	CBS 142401	MF795756	MF795756	MF795886	MF795798	
C. berberidis	CBS 130007[T]	KC506793	LT717673	LT717676	LT854936	
Elongatopedicellata aquatica	**FMR 17834 CBS 148920** [T]	**ON008448**				
E. lignicola	MFLUCC 15-0642 [T]	KX421368				
Flabellascoma aquaticum	KUMCC 15-0258 [T]	NG_068307	NR_166305		MN328895	MN328898

F. fusiforme	MFLUCC 18-1019	MT627660	MT627741		MT878452	MT95440
Fouskomenomyces cupreorufescens	A20 ^{T}	AY616236	AY616232			
F. mimiticus	FMR 16958 ^{T}	LR824585	LR824586			LR824584
Leptosphaeria dolium	CBS 125979	JF740283	JF740208			
L. dolium	CBS 505.75 ^{T}	GQ387576	JF740205			GU349069
Lophiostoma crenatum	CBS 629.86 ^{T}	DQ678069			KT216552	DQ677912
L. macrostomum	KT 635				JN993484	
L. semiliberum	KT 828	AB619014	JN942970		JQ012895	LC001759
Murispora aquatica	MFLU 19-0990 ^{T}	MN325075	MN325085			MN337969
M. cicognanii	MFLUCC 14-0953 ^{T}	NG_059609	NR_155381			MK109804
M. fagicola	MFLUCC 13-0600 ^{T}	NG_060797	NR_155379			KT709188
M. fissilispora	FMR 17151 ^{T}	LR824597	LR824594			LR824591
M. haswksworthii	MFLUCC 14-0918 ^{T}	KT709180	NR_138414			KT709192
M. medicaginicola	MFLUCC 13-0762 ^{T}	NG_059609	NR_155380			KT709191
M. rubicunda	IFRD 2017 ^{T}	FJ795507				GU456289
Neocucurbitaria acerina	MFLUCC 16-1450 ^{T}	NG_059784	NR_154254			
N. aquatica	CBS 297.74 ^{T}	EU754177	LT623221	LT623238	LT623278	
N. quercina	CBS 115095 ^{T}	GQ387619	LT623220	LT623237	LT623277	
N. unguis hominis	CBS 111112	GQ387623	LT623222	LT623239	LT623279	
N. unguis hominis	UTHSC: DI16-213 ^{T}		LT717672	LT717675	LT717682	
Neopyrenochaeta acicola	CBS 812.95 ^{T}	GQ387602	LT623218	LT623232	LT623271	
N. annellidica	MFLU 11-1105 ^{T}	MT183502	MT185538			
N. inflorescentiae	CBS 119222 ^{T}	EU552153	EU552153	LT623233	LT623272	

N. thailandica	MFLU 17-1461^{T}	NG_068716	MT214376		MT235825	
Neovaginatispora aquadulcis	**FMR 18914**<br>**CBS 148921**^{T}	**ON008451**	**ON006463**		**ON101724**	**ON101727**
N. clematidis	MFLU 17-2149^{T}	NG_073846	MT310606			MT394738
N. fuckelii	SICAUCC 20-0008	MT427734	MT427731	MT441924		MT441923
Pilidium acerinum	CBS 736.68^{T}	MH870939	NR_119500			
P. anglicum	CBS 143402^{T}	NG_058522	NR_156670			
P. cuprensens	**FMR 17839**<br>**CBS 148922**^{T}	**ON008449**	**ON006459**			
P. eucalyptorum	CPC:26594^{T}	NG_059618	NR_145311			
P. lythri	BPI 1107275^{T}	AY487095	AY487094			
P. lythri	BJ-4	MH322002	MH322003			
P. novae zelandiae	CPC:35872^{T}	NG_068665	NR_165928			
P. pseudoconcavum	CBS 136433^{T}	NG_058050	NR_137128			
P. septatum	BCC 79016^{T}	NG_060185	NR_156616			
Platystomum scabridisporum	BC 2235	GQ925844			GU479830	GU479857
P. scabridisporum	BC 2236	GQ925845			GU479829	GU479856
Pleospora herbarum	CBS 191.86^{T}	JX681120	NR_111243		KC584471	
P. typhicola	CBS 132.69	JF740325		KT389843	KC584505	
Pseudolophiostoma obtusisporum	KT 2838^{T}	LC312548	LC312518			LC312576
P. tropicum	KT 3134^{T}	LC312551	LC312522		LC312609	LC312580
Pseudopyrenochaeta lycopersici	CBS 306.65^{T}	EU754205	NR_103581	LT717674	LT717680	
P. terrestris	CBS 282.72^{T}	LT623216	LT623228	LT623246	LT623287	
Pseudosigmoidea alnicola	CBS 145034^{T}	NG_070465	NR_163379			

P. excentricum	CBS 469.95 [T]	MH874174	MH862538		
P. ibarakiensis	NBRC 107891 [T]	LC146759	LC146758		
P. robusta	**FMR 17416**	**ON008447**	**ON006458**		
Pyrenochaeta nobilis	CBS 407.76[T]	EU754206	EU930011	KT389845	LT623276
Pyrenochaetopsis americana	UTHSC: DI16-225[T]	LN907368	LT592912	LT592981	LT593050
P. aquatica	FMR 17327	LR216649	LR216648	LR897788	LR216647
P. botulispora	UTHSC: DI16-298	LN907432	LT592941	LT593010	LT593080
P. chromolaenae	MFLU 17-1446 [T]	MT214469	NR_168876		MT235824
P. confluens	CBS 142459[T]	LN907446	LT592950	LT593019	LT593089
P. cylindrispora	**FMR 18801**<br>**CBS 148924**	**ON008446**	**ON006461**	**ON101729**	**ON101725**
P. decipiens	CBS 343.85[T]	GQ387624	LT623223	LT623240	LT623280
P. globosa	UTHSC: DI16-275[T]	LN907418	LT592934	LT593003	LT593072
P. indica	CBS 124454[T]	GQ387626	LT623224	LT623241	LT623281
P. kuksensis	CBS 146534	MT371397	NR_172539	MT372662	MT372656
P. leptospora	CBS 101635[T]	GQ387627	JF740262	LT623242	LT623282
P. microspora	UTHSC: DI16-198	LN907341	LT592899	LT592968	LT593037
P. paucisetosa	UTHSC: DI16-193[T]	LN907336	LT592897	LT592966	LT593035
P. perfecta	**FMR 18913**<br>**CBS 148923** [T]	**ON008445**	**ON006462**	**ON101728**	**ON168928**
P. poae	CBS 136769[T]	KJ869175	KJ869117	KJ869243	LT623286
P. setosissima	CBS 119739[T]	GQ387632	LT623227	LT623245	LT623285
P. sinensis	LC12199	MK348581	MK348586	MK348221	MK355077
P. tabarestanensis	CBS 139506	KF803343	KF730241	KX789523	
P. terricola	HGUP1802	MH697393	MH697394	MH697392	MH697395

P. rajhradensis	CBS 146846 ^{T}	MT853182	MT853115	MT857726	MT857727	
P. uberiformis	UTHSC: DI16-277^{T}	LN907420	LT592935	LT593004	LT593074	
Roussoella arundinacea	CBS 146088	MT223928				
R. doimaesalongensis	MFLU 14-0584	NG_068241				
R. euonymi	CBS 143426 ^{T}	MH107961				
R. guttulata	MFLU 20-0102 ^{T}	MT734818				
R. hysteroides	CBS 546.94 ^{T}	MH874129				
R. intermedia	CBS 170.96 ^{T}	KF443382				
R. kunmingensis	HKAS 101773 ^{T}	MH453487				
R. margidorensis	MUT 5329	MN556322				
R. mediterranea	MUTITA 5369 ^{T}	MN556324				
R. mexicana	NTU 18-099-3	MT071282				
R. mukdahanensis	HKAS 101766	MH453485				
R. pustulans	KT1709 ^{T}	AB524623				
R. solani	KT 3265 ^{T}	LC195210				
Sporidesmium australiense	HKU 10833	DQ408554				
Sympoventuria capensis	CBS 120136 ^{T}	MH874632	NR_121323			
S. melaleucae	CBS 143407 ^{T}	NG_058520	NR_156668			
Torula herbarum	CBS 379.58	KF443383				
T. herbarum	CBS 111855	KF443386				
Tricladium splendens	CCM-F 16599	GQ477333	AY204635			
T. splendens	CBS 234.46 ^{T}	MH867695	MH856171			
Vaginatispora amygdali	KT 2248 ^{T}	LC312553	LC312524	LC312640	LC312611	LC312582
V. nypae	MFLUCC 18-1543 ^{T}	NG_066313	NR_163340		MK434877	MK360091
V. scabrispora	KT 2443 ^{T}	LC312554	LC312525	LC312641	LC312612	LC312583

Vargamyces aquaticus	CBS 636.91 [T]	KY853539	NR_154471
V. aquaticus	FMR 11587	KY853538	KY853475
Venturia inaequalis	CBS 814.69 [T]	MH871214	MH859439
V. pyrina	CBS 120825 [T]	MH874652	EU035468
Xeropilidium dennisii	TFC 201986 [T]		NR_171225
X. dennissi	KL159	KX090807	

[1]**A** Hermann Volgmayr; BC:; **BCC**: BIOTEC Culture Collection; **CBS**: Culture collection of the Westerdijk Biodiversity Institute, Utrech, The Netherlands; **CCM**: Czech Collections of Microorganims; **CPC**: Culture Collection of Pedro Crous; **FMR**: Facultat de Medicina, Reus, Spain; **HGUP**: Corresponding author's personal collection deposited in laboratory, housed at Guizhou, China; **HKAS**: Cryptogamic Herbarum of Kunming Institute of Botany, Chinese Academy of Sciences; **IFRD**: Culture Collection, International Fungal Research & Development Centre, Chinese Academy of Forestry, Kunming, China; **KT**: Kazuaki Tanaka; **KUMCC**: Kunming Institute of Botany Culture Collection; **LC**: Corresponding author's personal collection deposited in laboratory, housed at CAS, China; **MFLU (MFLUCC)**: Mae Fah Luang University Herbarum, Chiang Rai, Thailand; **MUT, MUTITA**: Mycoteca Universitatis Taurinensis; **NBRC**: NITE Biological Resource Center**; TFC**: Tartu Fungal Culture Collection; **UTHSC**, Fungus Testing Laboratory at the University of Texas Health Science Center, San Antonio, Texas, USA.
[2]Strains studied by us are indicated in **bold**.
[3]ITS: internal transcribed spacer region 1 and 2 including 5.8S nrDNA; LSU: large subunit of the nrRNA gene; *rpb2*: RNA polymerase II second subunit; *tub2*: ß-tubulin; *tef* 1: translation elongation factor.
[T]Ex-type strain.
